# Post-treatment with Telfairia occidentalis seed oil attenuates alcohol-induced testicular damage in Sprague-Dawley rats

**Published:** 2013-08

**Authors:** Ademola Ayodele Oremosu, Edidiong Nnamso Akang, Catherine Chukwumuanya Adigwe, Iniebehe Essien Okoko, Onyemaechi Okpara Azu

**Affiliations:** 1 *Department of Anatomy, College of Medicine, University of Lagos, Nigeria.*; 2 *Department of Anatomy, College of Medicine, Madonna University, Nigeria.*; 3 *Discipline of Clinical Anatomy, Nelson R Mandela School of Medicine, University of KwaZulu-Natal, Durban, South Africa.*

**Keywords:** *Telfairiaoccidentalis*, *Testis*, *Histology*, *Toxicity*, *Spermcount*

## Abstract

**Background:** Long term alcohol use has been implicated in men with sexual disorders including suppression of testosterone levels as well as testicular morphological changes.

**Objective:** This research investigated the ability of Telfairia occidentalis (T.O.) to attenuate the damaging effects of alcohol on the testicular parameters.

**Materials and Methods: **Thirty male Sprague-Dawley rats, 170-190 grams were divided into 6 groups, A to F and treated with distilled water (DW) for the period of 8 weeks (positive control group A), ethanol for 2 weeks followed with DW for 6 weeks (group B) (negative control), ethanol alone for 2 weeks (group C) while others received ethanol for 2 weeks, followed with 200 (group D), 400 (group E) and 600 mg/kg (group F) of T.O. for 6 weeks.

**Results:** Testicular histological sections showed that ethanol produced marked loss of testicular germ cells after two weeks of administration. T.O (200 mg/kg body weight) was not able to attenuate this microanatomical distortion when compared with control groups, but at 400 mg/kg body weight, T.O reversed the ethanol`s effects with resultant significant increase in sperm count and motility (p<0.05), serum testosterone levels (p<0.05), and testicular weight (p<0.05). However, at 600 mg/kg dosage, there was marked depletion of testicular germ cells with atrophied seminiferous tubules and a decrease in semen parameters and testicular weight.

**Conclusion:** Our result suggests that T.O promotes the regeneration of testicular germ cells and improves semen quality at a certain critical dose. Hence, T.O has a potential of reversing ethanol induced testicular damage.

## Introduction

There has been increasing concerns on the declining male fertility rates in the past 3-5 decades (1, 2). Approximately 50% of cases of infertility are male factor related and it is estimated that majority of these infertile males are alcohol consumers especially within the Nigerian context (3-6). Literature is rife with reports that alcohol affects the hypothalamo-pituitary-gonadal axis, reducing the production of luteinizing hormone (LH) and invariably affecting the production of testosterone (7, 8). It has also been established that alcohol increases the level of free radicals causing an elevation in oxidative stress hence impacting on the process of sperm production (9, 10).

Today, there is a renaissance on the use of medicinal herbs in the treatment of various ailments (11). The plant *Telfairia occidentalis* (T.O.) belonging to the cucurbitaceae family has been shown to have antibacterial, anti-inflammatory activities (12, 13). It is grown across the low-land and humid tropics in Western Africa (Nigeria, Ghana, Sierra leone being the major producers) (14). In Nigeria, the herbal preparation of the plant has been employed in the treatment of anaemia, chronic fatigue, diabetes, and enhancing sperm quality (15-18). 

T.O. is normally grown in the tropics and is widely consumed in the South-South region and South-Eastern parts of Nigeria (18). The seed is rich in vitamins A, C and E. It also contains iron, magnesium, potassium, phenols, carotenes, and fatty acids (12). The unsaturated fatty acids are composed mainly of oleic (61.83%) and linoleic (16.44%) while the saturated fatty acids mainly of palmitic acid (21.20%) (19). Vitamins C and E are strong antioxidants and have been used to protect against free radicals and reduce lipid peroxidation (20). T.O seed is also rich in flavonoids which have been used in the treatment of reproductive endocrine diseases in men and in women (21). Flavonoids also have profound effects on semen quality and the functionality of the accessory sex organs (22).

The amino acid profile of T.O. had also been shown to be very rich and includes alanine, aspartate, glycine, glutamine, histidine, lysine, methionine, tryptophan, cystine, leucine, arginine, serine, threonine, phenylalanine, valine, tyrosine, and isoleucine (23). We have reported that prophylactic treatment with T.O protects the testes from the ravages of ethanol but the consequences of acute alcohol abuse and the probable ameliorative effects of post-treatment with T.O on testicular reproductive parameters remains to be investigated (24). Therefore, this work is set out to fill this gap in knowledge.

## Materials and methods

**Extract preparation**

Healthy fruits of T.O were obtained from Mushin market, Lagos during the month of June, 2009. It was authenticated in the Department of Botany and Microbiology, University of Lagos where a voucher number-LUH 2763 was issued for ease of future reference. A large quantity of naked fluted pumpkin seeds were dried in the oven in the laboratory at 30^o^C for 48 hours. 

The seeds were blended to a fine powder using Maurine electric blender and preserved for extraction of the oil samples. The oil extract was obtained using petroleum ether in continuous extraction in a Soxhlet-type extractor as described by Agatemor (25). At the completion of extraction, the petroleum ether was completely evaporated using a rotary evaporator at 35^o^C (Heidolph Laborota 4010 digital). The concentrated oil was the desired sample. 

**Treatment and dosage**

This was an experimental animal study and ethanol was administered orally at a dose of 2 g/kg body weight and a concentration of 30% volume/volume while T.O seed oil was administered intraperitoneally at 3 different doses of 200 mg/kg, 400 mg/kg and 600 mg/kg body weight (10, 24).

**Design**

Thirty healthy male Sprague-Dawley rats weighing 170-190 grams were procured from the Laboratory Animal Centre of the College of Medicine, University of Lagos. The rats were fed on standard diet (Bendel Feed and Flour Mills Ltd); had access to water ad libitum and were maintained under standard conditions. The animal room was well ventilated, maintained in a temperature range of 25-28^o^C under day/night 12-12 h photoperiodicity. The rats were randomly divided into six groups of 5 rats each (A, B, C, D, E, and F). All procedures guiding the use of the animals were in accordance with the standard international guidelines on the use of animals for research. 

Approval for the study was obtained from the Departmental Ethics Committee and also granted by the Experimental Ethics Committee on Animals Use of College of Medicine, University of Lagos, Nigeria. Group A (positive (+ve control) received distilled water for 8 weeks. Group B (negative (-ve control) received 30% v/v of ethanol for 2 weeks followed by distilled water for 6 weeks. Group C received 30% v/v of ethanol for 2 weeks only. Groups D, E and F received 30% v/v of ethanol for 2 weeks followed with 200, 400 and 600 mg/kg bodyweight (bw) of T.O respectively for 6 weeks. 

**Organ harvesting**

At the end of experimental period, blood was collected from the medial canthus of the eye into plain sample bottles and centrifuged at 3000 rpm for 10 minutes to obtain clear sera for hormonal assay. The rats were sacrificed by cervical dislocation and placed on dorsal recumbence. The testes were surgically removed through a lower abdominal incision. It was trimmed of fats, weighed and processed for histological examination. The epididymides were excised; several small cuts were made in the cauda epididymis and suspended in 1ml of normal saline for the spermatozoa to swim up and samples were taken for semen analysis 

**Histological preparation of tissues**

Histological processing of the testes was done as described in Azu *et al* (26). Briefly, the testes were harvested and fixed in 10% buffered formalin for 24 hours after which they were dehydrated in graded alcohol concentrations and cleared in xylene before infiltration in molten paraffin wax and embedded. Serial sections were cut using the rotary microtome at 6 microns thickness. Sections were then processed and stained with routine H&E and viewed with the Olympus® microscope at magnification of X400. 

**Testicular weight (TW)**

TW were taken before histological processing using Scout (tm) Pro SPU 2001, electronic weighing balance, manufactured by Ohaus Corporation, Pine Brook, NJ USA.

**Hormonal assay**

The blood specimens from the subjects were collected into plain sample bottles and were immediately centrifuged to separate the sera from the cells. The sera were labelled and analysed. Testosterone and luteinizing hormones were assayed using the enzyme immunoassay methods of Diagnostic Automation, Inc. (2008).

**Sperm motility analysis**

The slides on which the sperm cells were counted were warmed to 37^o^C until the time of the analysis. The analysis was carried out at room temperature using one cauda epididymis of each rat. The percentage of sperm motility was calculated using the average of the number of live sperm cells with forward movement over the total number of sperm cells (both motile and non-motile), from two samples from one cauda epididymis of each rat (27).

**Sperm count**

This was determined using the new improved Neubauer`s counting chamber (Haemocytometer). The epididymal fluid from both cauda epididymides was diluted with physiological solution by adding 0.9 ml to 0.1 ml of the crushed epididymides. The counting chamber was charged with a cover slip until a rainbow picture was seen at the edges. This chamber was then filled with sperm fluid and placed under a binocular light microscope using an adjustable light source. The ruled part was then focused and the number of spermatozoa counted in five 16-celled squares. The total sperm cells were added, divided by two and multiplied by 10^6^ and expressed as (X) ×10^6^/ml, where X is the total number of sperm cells in the five 16-celled square after dividing by two (28).

**Statistical analysis**

The data obtained from all the groups were compiled and statistically analyzed and expressed as mean±SD. Since we had a parametric data, differences between groups were compared using One-way ANOVA, with p<0.05 considered significant.

## Results

**Testicular weight**

There was no significant difference in TW in all the experimental animals except for the group that received 2 weeks of ethanol and 6 weeks of 400 mg/kg of T.O that showed a significant increase (0.05). Although group C animals that received 2 weeks of ethanol showed an increase in TW, it was not significant when compared with controls (0.05) (Table I). 

**Semen parameters**

Sperm count and motility were significantly increased in group E treated animals when compared with positive control (0.05). Ethanol alone group (group C) also recorded a significantly decreased sperm motility at 31.7±7.6% (0.05) but the sperm count was not significantly so. The animals that received 200 mg/kg of T.O and ethanol (group D) and 600 mg/kg of T.O and ethanol (group F) showed seminal parameters that were close to those of negative controls (Table I). 

**Hormonal assay**

There was a statistically significant increase in serum testosterone levels of animals that received 2 weeks of 30% v/v ethanol and 400 mg/kg b.w of T.O for 6 weeks (group E) compared with negative control (group B) (0.05). The animals that received ethanol for 2 weeks and those that received ethanol followed with 200 mg/kg of T.O (groups C and D respectively) showed a significant decrease in serum testosterone when compared with positive control (group A) but animals that received ethanol followed with 600 mg/kg showed a decrease which was not statistically significant when compared with controls. Though LH values in groups C, D and E were lower than those of controls and group F slightly higher than positive control, these values were not significant (0.05) (Table II).

**Testicular histology**

**Figure 1:**
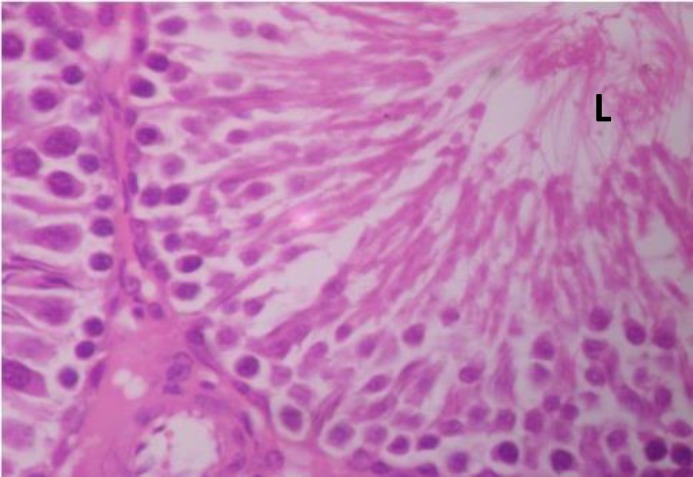
Testicular cross section group A (+ ve control) showing normal histo-architecture. H&E×400. L- lumen containing sperm cells.

**Figure 2:**
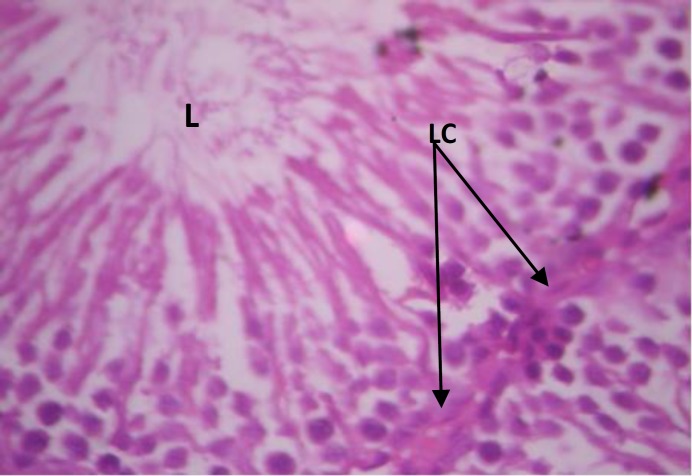
Testicular cross section group B animals (-ve control) H&E ×400; SS- Spermatogenic cell series, LC- Leydig cells, L- sperm cells in lumen

**Figure 3:**
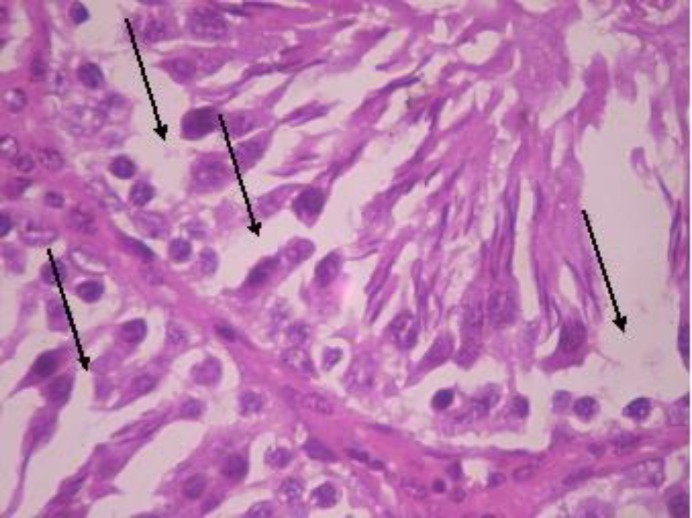
Testicular cross section group C animals showing depleted spermatogenesis. H&E ×400. Arrows shows areas of marked depletion of spermatogenic cells

**Figure 4:**
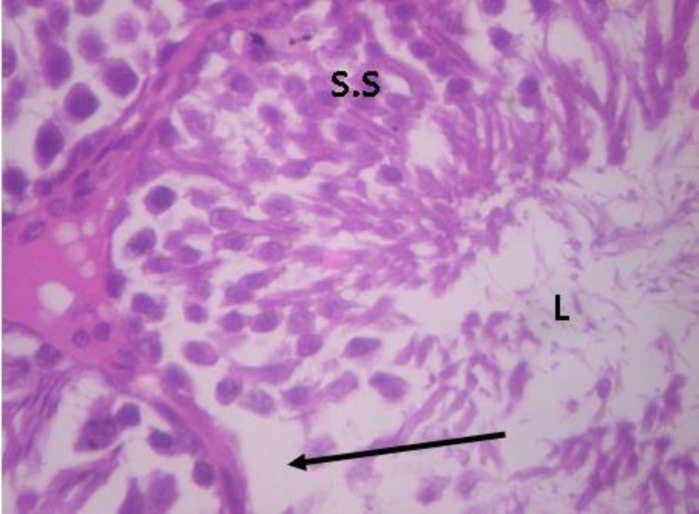
Testicular cross section of group D animals showing areas of focal area of spermatogenic depletion (arrowed). H&E ×400. S.S- well arranged Spermatogenic cell series; L sperm cells in lumen of seminiferous tubule

Testicular histopathology of control groups showed normal architecture and cellular composition with no obvious aberrations (Figures 1 and 2). There was marked depletion of spermatogenic cells (mostly the primary and secondary spermatocytes) in group C animals treated with ethanol for 2 weeks (Figure 3). In group D testicular sections showed some spermatogenic cell series with few degradation of cellular composition and a normal interstitium (Figure 4). 

**Figure 5:**
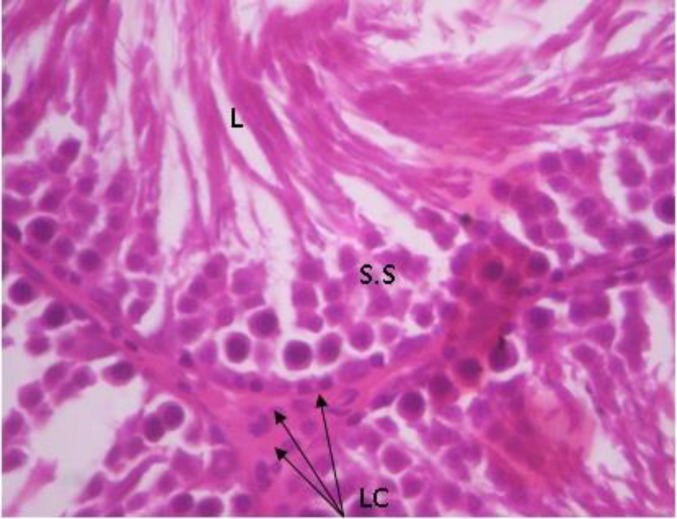
Testicular cross section of group Eanimals with normal microanatomical display. H&E ×400. S.S spermatogenic cell series. LC-Leydig cells, L- sperm cells in lumen.

**Figure 6:**
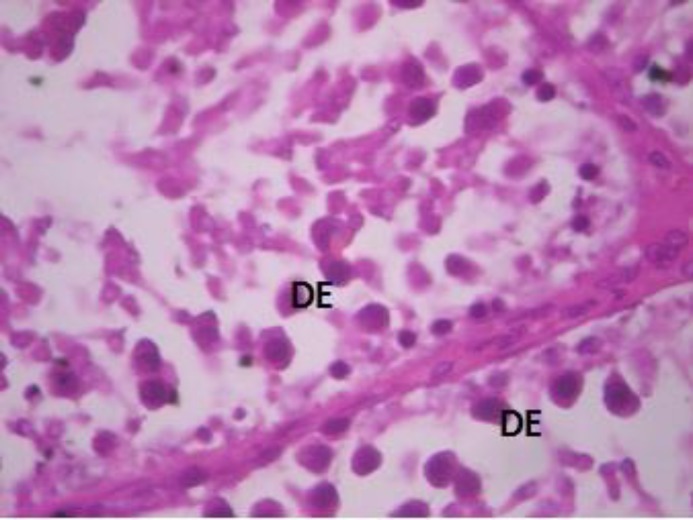
Testicular cross section of group F animals with distorted epithelium. H&E×400. DE- distorted seminiferous tubules

Testicular sections of group E animals were essentially normal and similar to controls with all cells of the spermatogenic series and normal interstitium (Figure 5). Group F animals showed seminiferous tubules that were devoid of the normal architectural pattern with complete absence of spermatogonia in many of the seminiferous tubules and atrophy of tubules (Figure 6).

**Table I:** Effect of Telfairia occidentalis on sperm count, sperm motility and testicular weight

**Groups**	**Treatment administered **	**Motility %**	**Count (10** ^6^ **/ml)**	**Testicular weight (g)**	
A	Distilled water 8 wks	55.8±4.3	40.3±9.5	0.9±0.1	
B	Ethanol 2 wks, distilled water 6 wks	46.7±2.9	51.7±2.9	0.9±0.1	
C	Ethanol 2 wks	31.7±7.6**	48.3±2.9	1.1±0.1	
D	Ethanol 2 wks, FPSO (200mg) 6 wks	48.3±7.6	52.3±2.5	0.8±0.1	
E	Ethanol 2 wks, FPSO (400mg) 6 wks	63.3±5.8*	59.7±9.1*	1.4±0.4*	
F	Ethanol 2 wks, FPSO (600mg) 6 wks	46.7±5.8	40.3±12.7	0.8±0.2	

Wks: weeks

FPSO: Fluted pumpkin seed oil

Values are mean ± standard deviation. One-way ANOVA

* p<0.05 vs. + ve control group (A).

** p<0.05 vs. - ve control group (B)

**Table II:** Effect of T.O on serum testosterone and luteinizing hormones

**Groups**	**Treatment administered**	**Testosterone (ng/ml)**	**Luteinizing hormone (ng/ml)**
A (+ve control)	Distilled water 8 wks	3.0±1.9	3.3±1.2
B (-ve control)	Ethanol 2 wks,distilled water 6 wks	1.8±0.2	2.9±0.8
C	Ethanol 2 wks	1.1±0.1*	2.0±0.2
D	Ethanol 2 wks, FPSO (200mg) 6 wks	1.3±0.4*	1.8±0.5
E	Ethanol 2 wks, FPSO (400mg) 6 wks	2.8±0.3**	1.7±0.2
F	Ethanol 2 wks, FPSO (600mg) 6 wks	1.6±0.2	3.4±0.6

Wks: weeks

FPSO: Fluted pumpkin seed oil

Values are mean ± standard deviation. One-way ANOVA

* p<0.05 vs. + ve control group.

** p<0.05 vs.- ve control group

## Discussion

The present study has shown that administration of seed extract of T.O at a dose of 400 mg/kg bw was able to attenuate the ravages of ethanol-induced injuries and caused significant increase in testosterone levels, increased overall testicular weight as well as improved sperm count and motility during the experimental period. This result agrees with our previous finding (24, 29) where T.O at similar dose improved sperm parameters. 

The present study has also confirmed that the 400 mg/kg dose of T.O seems to produce best results as against higher or lower doses applied. An explanation for this might not be unconnected with the chemical composition of T.O which has been shown to be rich in active ingredients (Vitamin C and E, flavonoids, oleic acid and linoleic acid) documented to have stimulatory spermatogenic activities (30). T.O seed is highly nutritious thus providing additional nutrients to the treated animals by way of carbohydrates, fats and proteins (11). 

arbohydrates provide energy-rich source for pyruvates needed for sperm survival the amino acid arginine is an important precursor for spermine needed for sperm motility and ascorbic acid is reported to improve sperm motility (31-34). 

Ethanol administration reduced not only serum testosterone level but also other sperm parameters (count and motility) with accompanied low LH levels. This reduction in the serum testosterone could be due to decreased synthesis as postulated by Manesh *et al* (35). 

As expected, decline in testosterone levels is accompanied by a coordinated increase in levels of LH and FSH to stimulate the production of more testosterone (36). But in our study we found that low serum testosterone level in the alcohol-treated animals was accompanied by low serum LH levels. This finding suggests that the hypothalamic cells, which produce luteinizing hormone releasing hormone (LHRH), do not function correctly to the feedback when testosterone level decreased. A clearer picture of what is happening at the cellular level of the seminiferous tubules mirrored these perturbations in hormonal levels; they were concomitant marked depletion of sperm cells. 

These results are similar to the pattern of derangement in toxic drug injuries to the Sertoli cells creating disruptions in the interdigitations of the tight barriers necessary for the functionality of the seminiferous epithelium as has been described in Azu *et al* (37). Although testicular oxidative stress markers was not estimated in this study, alcohol is known to cause enormous systemic oxidative stress marked by increased serum level of oxidizing agents and decreased serum level of potential scavengers of reactive oxygen species (i.e. antioxidants). Hence, increased oxidative stress is a well-accepted mechanism of alcohol induced tissue injury and this also occurs in the testes (35, 38). 

The assumption that free radicals can influence male infertility has received substantial scientific support (39, 40). T.O is rich in free radical scavengers (vitamins C, E) and phytochemicals (phenols). Among many classes of compounds, phenolics have been recognised as a powerful counter measure against lipid peroxidation in biologic systems by scavenging free radicals and quenching the lipid peroxidative side chains via their hydrogen-donating ability (37). We believe that probably, T.O is able to attenuate the deleterious effects of alcohol on the testis via this mechanism. 

Moreover, oleic acid and vitamin A which is present in the seed oil has been reported to reduce lipid peroxidation (41-43). In view of the fact that alcohol consumption has become increasingly recreational and the long term consequences of these are unsavory resulting to impotence, testicular atrophy and loss of sexual interest in men, our findings on the post-treatment effects of T.O seed oil has a significant positive milestone in the continued quest for a readily-available, natural source for the treatment of male-factor infertility (5, 44). From the histological standpoint, post-treatment with T.O following ethanol-induced damage on the testes were restored by 400 mg/kg dose of T.O evidenced by histo-architectural patterns that were similar to positive controls. 

Looking at the distortions caused by ethanol exposure in the various treatment groups and the level of damage-control offered by T.O, it can be safe to conclude that the qualitative histological changes were restored by T.O at the dose of 400 mg/kg as against the other tested treatment dose regime. This observation is mirrored by other parameters (spermatogenic and hormonal) that were recorded. But the clear explanation as to why lower or higher doses of T.O were not as effective as the 400mg/kg dosage remains elusive, although iron has been implicated as a possible culprit in the pro-oxidant function at high doses as we earlier adduced (24).

Our result further supports that the circulating level of androgen was enough to maintain testicular weight which is in agreement with the fact that the structural and functional integrity of reproductive tissues depends on the circulating androgens (37). While the administration of 400 mg/kg T.O for 42 days post-ethanol brought about an increase in testicular weight as against declines in negative controls, this was also supported by the positive values for testosterone and sperm motility further supporting the fact that alcohol produces a significant decrease in sperm motility and morphology (45). 

The epididymis is an important structural component of the testis conferring fertility ability on the spermatozoa and the development of rapid progressive movement. The spermatozoa recovered from the cauda epididymis of the rats after 6 weeks post-treatment with 400 mg/kg T.O were 63.3% motile against 31.7% in ethanol alone treated animals or 55.8% in positive control. These sperms are capable of forward movement needed for fertilisation and this further indicates that there might be no defects in flagellar substructures (37). 

In this study, the negative control group showed a good recovery in sperm count and motility although histological assessment and hormonal parameters did not show a complete reversal to positive control state after withdrawal of ethanol at the stated duration. This is similar to what Martinez *et al*, Van Thiel *et al* and Anderson *et al* had previously reported in their work; the recovery of the various reproductive variables of the testis and other androgen-dependent organs after alcohol withdrawal may not be ruled out in this study (46-48). 

## Conclusion

Ethanol administration resulted in deleterious histological testicular changes, low plasma testosterone with concomitant low LH levels in this study. T.O at a dose of 400 mg/kg post-treatment attenuated the damaging effects of ethanol mirrored by restoration in sperm parameters, hormonal assessment and qualitative histological assessment. Further work is needed to establish the precise stereological indices, stress-status and specific active components in T.O with a view to understand the actual mechanism of action of T.O. 

## Conflict of interest

Authors received nor financial grants or support for this study. The authors declare that there are no conflicts of interest in the work.

## References

[1] Kumar R, Venkatesh S, Kumar M, Tanwar M, Shasmsi MB, Kumar R (2009). Oxidative stress and sperm mitochondrial DNA mutation in idiopathic oligoasthenozoospermic men. Indian J Biochem Biophys.

[2] Saalu LC, Osinubi AA (2009). Environmental Endocrine Disruptors of Testicular Function. Afr J Endocrinol Metabol.

[3] Sharlip ID, Jarow JP, Belker AM, Lipshultz LI, Sigman M, Thomas AJ (2002). Best practice policies for male infertility. Fertil Steril.

[4] Onyeka CA, Ashiru OA, Duru FI, Olurunfemi OJ, Fabunmi OO, Oluwatuyi TS Semen analysis of 263 sample men from infertility clinic in Western Nigeria.

[5] Okonofua F, Menakaya U, Onemu SO, Omo-Aghoja LO, Bergstrom S (2005). A case-control study of risk factors for male infertility in Nigeria. Asian J Androl.

[6] Agarwal A, Sharma RK, Nallella KP, Thomas AJ Jr, Alvarez JG, Sikka SC (2006). Reactive oxygen species as an independent marker of male factor infertility. Fertil Steril.

[7] Little PJ, Adams ML, Cicero TJ (1992). Effects of alcohol on the hypothalamic-pituitary-gonadal axis in the developing male rat. J Phamacol Exp Ther.

[8] Emanuele M, Emanuele N (2001). Alcohol and male reproductive system. Alcohol Res Health.

[9] Taati M, Alirezaei M, Meshkatalsadat MH, Rasoulian B, Kheradmand A, Neamati Sh (2011). Antioxidant effects of aqueous fruit extract of Ziziphus jujuba on ethanol-induced oxidative stress in the rat testes. Iran J Vet Res Shiraz Univ.

[10] Dosumu OO, Akinola BO, Akang EN (2012). Alcohol-induced testicular oxidative stress and cholesterol homeostasis in rats- The therapeutic potential of virgin coconut oil. Mid East Fertil Soc J.

[11] Kayode AAA, Kayode OT (2011). Some Medicinal Values of Telfairia occidentalis: A Review. Am J Biochem Mol Biol.

[12] Odoemena CS, Onyeneke EC ( 1998). Lipids of fluted pumpkin (Telfairia occidentalis) seeds.

[13] Oluwole FS, Falode AO, Ogundipe OO (2003). Anti-inflammatory effect of some common Nigeria vegetables. Nig J Physiol Sci.

[14] Nkang A, Omokaro D, Egbe A, Amanke G (2003). Variations in fatty acid proportions during desiccation of Telfairia occidentalis seeds harvested at physiological and agronomic maturity. Afr J Biotechnol.

[15] AderIbigbe AO, Lawal BAS, Oluwagbemi JO (1999). The antihyperglycaemic effect of Telfairia occidentalis in mice. Afr J Med Med Sci.

[16] Alada ARA (2000). The haematological effects of Telfairia occidentalis diet preparation. Afr J Biomed Res.

[17] Dina OA, Adedapo AA, Oyinloye OP, Saba AB (2000). Effect of Telfairia occidentalis extract on experimentally induced anaemia in domestic. Afr J Biomed Res.

[18] Salman TM, Olayaki LA, Oyeyemi WA (2008). Aqueous extract of Telfairia occidentalis leaves reduces blood sugar and increases haematological and reproductive indices in male rats. Afr J Biotechnol.

[19] Akintayo T (1997). Chemical composition and physicochemical properties of fluted pumpkin (Telfairia occidentalis] seed and seed oils. CODEN RISGAD.

[20] Lee K, Dabrowski K (2004). Long-term effects and interactions of dietary vitamins C and E on growth and reproduction of yellow perch, Perca flavescens. Aquaculture.

[21] Qin D, She B, She1Y, Wang J (2000). Effects of flavonoids from Semen Cuscutae on the reproductive system in male rats. Asian J Androl.

[22] Das S, Parveen S, Kundra CP, Pereira BMJ (2004). Reproduction in Male Rats is Vulnerable to Treatment with the Flavonoid-rich Seed Extracts of Vitex negundo. Phytother Res.

[23] Tindall HD (1968). Commercial Vegetable Growing.

[24] Akang E, Oremosu A, Dosumu O, Ejiwunmi A (2011). Telfairia Occidentalis, a Prophylactic Medicine for Alcohol’s Damaging Effect on the Testis. Macedonian J Med Sci.

[25] Agatemor C (2006). Studies of selected phytochemical properties of flute dpumpkin (Telferia occidentalis hook F.) seed oil and tropical Almond (Terminalis catappia I) seed oil. Pak J Nutr.

[26] Azu OO, Duru FIO, Osinubi AA, Noronha CC, Elesha SO, Okanlawon AO (2010a). Preliminary study on the antioxidant effect of Kigelia africana fruit extract (Bignoniacieae) in male Sprague-Dawley rats. Afr J Biotech.

[27] Yan J, Agresti M, Bruce T, Yan Y, Granlund A, Matloub H (2007). Effects of cellular phone emissions on sperm motility in rats. Fertil Steril.

[28] Keel BA, Webster BW (1990). CRC Handbook of the Laboratory Diagnosis and Treatment of Infertility.

[29] Akang EN, Oremosu AA, Dosumu OO, Noronha CC, Okanlawon AO (2010). The effect of fluted pumpkin (Telferia occidentalis) seed oil (FPSO) on testis and semen parameters. Agr Biol J North Am.

[30] Amanvermez R, Serif D, Ozgur K, Tuncel Muhlise A, Agar E (2005). Alcohol-induced oxidative stress and reduction in oxidation by ascorbate /I-cys/I-met in the testis, ovary, kidney and lung of rat. Adv Ther.

[31] Egbunike GN, Branscheid W, Pfisterer J, Holtz W (1986). Changes in porcine sperm lactate dehydrogenase isoenzymes during sperm maturation. Andrologia.

[32] Morales ME, Rico G, Bravo C, Tapia R, Alvarez C, Méndez JD (2003). Progressive motility increase caused by L-arginine and polyamines in sperm from patients with idiopathic and diabetic asthenozoospermia. Ginecol Obstet Mex.

[33] Dawson EB, Harris WA, Teter MC, PowellLC (1992). Effects of ascorbic acid supplementation on the sperm quality of smokers. Fertil Steril.

[34] Fasuyi AO (2006). Nutritional potentials of some tropical vegetable leaf meals: Chemical characterization and functional properties. Afr J Biotechnol.

[35] Maneesh M, Jayalekshmi H, Sanjiba Dutta, Amit Chakrabarti, Vasudevan DM (2005). Experimental therapeutical intervention with ascorbic acid in ethanol induced testicular injuries in rats. Indian J Exp Biol.

[36] Cevik R, AH Gur, Suat Acar, Kemal Nas, AyÕ egül Jale Sarac (2004). Hypothalamic-pituitarygonadal axis hormones and cortisol in both menstrual phases of women with chronic fatigue syndrome and effect of depressive mood on these hormones. BMC Musculoskelet Disord.

[37] Azu OO, Duru FIO, Osinubi AA, Oremosu AA, Noronha CC, Elesha SO (2010b). Histomorphometric effects of Kigelia africana (Bignoniaceae) fruit extract on the testis following short-term treatment with cisplatin in male Sprague-Dawley rats. Mid East Fertil Soc J.

[38] Gagnon C, Iwaaski AO, de Lamirande E, Kovaski N (1991). Reactive oxygen species and human spermatozoa. Ann N X Acad Sci.

[39] Mari M, Wu D, Nieto N, Cederbaum AJI (2001). CYP2E1- Dependent Toxicity and Up-Regulation of Antioxidant Genes. J Biomed Sci.

[40] Tremellen K (2008). Oxidative stress and male infertility- a clinical perspective. Hum Reprod Update.

[41] Lovejoy JC (2002). The influence of dietary fats in insulin resistance. Cur Diabetes Reports.

[42] Bourre J, Dumont O, Durand G (2004). Dose-effect of dietary oleic acid: Oleic acid is conditionally essential for some organs. Reprod Nutr Dev.

[43] Patil RB, Vora SR, Pillai MM (2009). Antioxidant effect of plant extracts on phospholipids levels in oxidatively stressed male reproductive organs in mice. Iran J Reprod Med.

[44] Friedler G, Cicero TJ (1987). Paternal pregestational opiate exposure in male mice: neuroendocrine deficits in their offspring Res. Commun Subst Abuse.

[45] Donnelly GP, McClure N, Kennedy MS, Lewis SE (1999). Direct effect of alcohol on the motility and morphology of human spermatozoa. Andrologia.

[46] Martinez FE, Martinez M, Cagnon VHA, Mello Junior W, Padovani CR, Garcia PJ (1997). Effects Of experimental chronic alcoholism on the seminal vesicle and testis weight of adult rats (Rattus norvegicus). Rev Chil Anat.

[47] Van-Thiel DH, Gavaler JS, Sangavi A (1982). Recovery of sexual function in abstinent alcoholic men. Gastroenterology.

[48] Anderson RA, Willis BR, Oswald C (1985). Partial reversal of ethanol-induce male reproductive pathology following abstinence. Alc Alc.

